# Exercise-Induced Rhabdomyolysis: A Case Series of Spin-Related Rhabdomyolysis

**DOI:** 10.7759/cureus.16352

**Published:** 2021-07-13

**Authors:** Lina Pei Shi Yow, Han Yao Ho, Isaac Yong Wai Lum, Ibrahim M Hanif

**Affiliations:** 1 Internal Medicine, Sengkang General Hospital, Singapore, SGP

**Keywords:** exertional rhabdomyolysis, indoor cycling, exercise physiology, exercise training, sports injury, spin

## Abstract

Exercise rhabdomyolysis is a potentially life-threatening medical condition if not adequately managed early. With the increase in the popularity of indoor cycling, known as Spinning®, over recent years, there has been an increased occurrence of spin-related rhabdomyolysis observed among previously fit adults after undertaking their first spin bike class session.

They present with the triad of myalgia, muscle weakness, and dark tea-colored urine within a week of their first spin session. This case series highlights several admissions to the hospital with spin-related rhabdomyolysis and their clinical management.

## Introduction

Indoor cycling, known as Spinning®, has gained an increase in popularity over the last decade. With the current coronavirus disease 2019 (COVID 2019) pandemic and lack of ability for air travel, there has been a general increase in the number of people undertaking new sporting activities in Singapore, particularly spinning, as there is a sudden urge to get back on track with fitness or pick up a new hobby.

However, over the last two years (spanning the COVID pandemic), the general medicine department in our local regional hospital has observed an increase in the number of rhabdomyolysis admissions attributed to spin classes. On the other hand, we have not observed a similar increase in exercise-related rhabdomyolysis particularly attributed to other sporting activities. We present five cases of spin-associated rhabdomyolysis and reflect on the lesson learned in managing these cases, in order to bring awareness to the medical profession as well as the public.

These patients were all virgin spin attendees of a 1-hour spin class. The commonality amongst the five cases was that of individuals with no past medical history, no medication or over-the-counter or traditional medication use, no illicit drug or steroid use, no intercurrent infective symptoms, no history of trauma or falls, and no family history of neurological disease.

## Case presentation

Case number: 1

A 29-year-old male presented to the emergency department with a three-day history of bilateral thigh pain following a spin class. This was associated with weakness of the bilateral lower limbs and the passing of dark-colored urine. He took paracetamol with no effect.

On examination, the significant findings were bilateral thigh tenderness and proximal weakness of bilateral lower limbs. The proximal power of bilateral lower limbs was 3-4 out of 5 while the distal power remained at 5 out of 5.

He was admitted for six days and administered a daily 3L intravenous infusion of Hartmann’s solution management. The bilateral thigh pain and proximal lower limb weakness improved together with the creatine kinase (CK) level. The CK level prior to discharge was 1933 U/L. He was reviewed in the clinic two weeks post-discharge and by then the CK level and liver function test had completely resolved.

Case number: 2

A 31-year-old male presented with a two-day history of bilateral thigh pain and darkening of urine following a spin session. He does regular gym workouts two to three times a week but was spin naive. The bilateral thigh pain was graded 8 out of 10 on the pain scale, with associated bilateral lower limb weakness and reduced range of knee motion. He noted the darkening of his urine on the second-day post-spin session. He denies any lower urinary tract symptoms. He reported having drunk two pints of beer immediately after the spin session.

On examination, there was bilateral thigh tenderness on palpation, with proximal weakness of 4 out of 5 in the bilateral lower limb. The power of the distal lower limb was 5 out of 5 bilaterally. The range of movement of the right knee was 0-90 but the left knee was 0-60. There was no joint tenderness or effusion over bilateral knees.

The patient was given a 3L intravenous Hartmann’s solution daily. The patient was discharged on the third day because the patient was keen for going home in view that his thigh pain and lower limb weakness had resolved by then. His CK level was still elevated at 31738 U/L prior to discharge. He was encouraged to have an oral fluid intake of 2-3L daily. He was reviewed in the clinic one-week post-discharge, and his CK level was 583U/L. The CK level subsequently normalized the following week.

Case number: 3

This patient is a 30-year-old lady who presented to the emergency department with a two-day history of bilateral thigh pain. The thigh pain and cramps started one day post-spin session. She does not exercise regularly, with only one to two exercise sessions of moderate-intensity per month at most.

Significant findings on examination were that of bilateral thigh tenderness on palpation with limited range of motion of bilateral knees of 0-70 degrees, with proximal weakness bilateral of 3 out of 5. The power of distal lower limbs was 4+/5. There was no joint tenderness or swelling over the knees.

The patient was initially started on a daily 2.5L intravenous infusion with Hartmann’s solution. However, the CK continued to trend upwards by the third day of admission. Hence, the decision was made to add 0.5L of isotonic bicarbonate intravenous infusion to the regimen. The CK level started to downtrend on Day 5 of admission. The liver transaminases were elevated with aspartate transaminase (AST) being more than alanine transaminase (ALT) in keeping with rhabdomyolysis.

There was an associated improvement in liver enzymes with a downward trend in CK. The patient’s bilateral thigh pain subsequently improved with the recovery of a full range of motion of bilateral knees and she was discharged on Day 9 of admission. She was reviewed in the clinic six days later, CK levels continued to improve to 659 U/L and subsequently normalized.

Case number: 4

The patient is a 28-year-old male who presented to the emergency department with a two-day history of bilateral thigh pain, having had attended his first spin class four days prior. He also went to do bungee jumping two days after the spin but did not report any trauma or falls during bungee jumping. He noticed tea-colored urine and bilateral thigh pain one day after the bungee jumping.

On examination, the significant findings were bilateral thigh tenderness and proximal weakness. The rest of the physical examination was unremarkable. The proximal power of bilateral lower limbs was 3-4 out of 5 while the distal power remained at 5 out of 5.

The patient was given a 3L intravenous infusion with Hartmann’s solution on admission. CK levels continued to rise to > 100000 on the fourth day of admission. Subsequently, CK levels started decreasing on the fifth day of admission, and on Day 7 of admission, it was 12399 U/L. With the improvement of CK levels, the patient’s symptoms of thigh pain and weakness of the lower limbs also improved. The patient was discharged on Day 7 of admission.

He was reviewed in the clinic one week post-discharge and the CK level was 445. The AST and ALT have normalized to be within range.

Case number: 5

A 33-year-old lady was admitted with worsening pain over bilateral thighs and darkening of urine over three days duration since her spin class. The patient was running or cycling at moderate intensity once a week prior to the spin class.

On examination, there was bilateral thigh tenderness on palpation associated with a limited range of motion of bilateral knees. The right knee range of motion 0-90 degrees, left knee range of motion 0-110 degrees. There was a proximal weakness of bilateral lower limbs 4/5.

She was started on daily intravenous hydration with 2.5L Hartmann’s solution and 0.5L isotonic sodium bicarbonate. The CK levels subsequently down-trended on Day 2 of admission. This was also associated with improvement in AST and ALT. The patient was also able to ambulate independently by self with improved pain and regained full range of motion of knee bilaterally. The patient was discharged on Day 5 of admission and encouraged to continue oral hydration. She was reviewed in the clinic five days post-discharge; CK levels were 579 U/L.

Table 1 summarizes the laboratory results.

**Table 1:** Summary of laboratory results of presented cases WBC: White Blood Cell; ALT: Alanine Transaminase; AST: Aspartate Transaminase; ALP: Alkaline Phosphatase; GGT: Gamma-Glutamyl Transferase

	Patient 1	Patient 2	Patient 3		Patient 4			Patient 5
Gender	Male	Male	Female		Male			Female
Height (cm)	169	189	159		175			155
Weight (kg)	55.8	87.4	55		105.5			50.4
BMI	19.5	24.5	21.8		34.5			21
Creatine Kinase (44 – 201 U/L)								
On Admission	>100 000	42 983	24 789		62 280			50 278
On Discharge	1933	31 738 (rechecked 1 week later: 583 U/L)	4268 (rechecked 1 week later: 697 U/L)		12 399 (rechecked 1 week later: 445 U/L)			7090 (rechecked 1 week later: 445 U/L)
Duration of admission	6 days	3 days	9 days		7 days			5 days
Full Blood Count								
Hemoglobin (12 – 16 g/dL)	15.9	15.3	14.0		17.3			13.3
WBC (4 – 10 x10^9^/L)	6.84	7.39	10.16		13.81			8.69
Platelet (140 – 440 x 10^9^/L)	278	238	415		314			212
Renal panel								
Urea (2.7 – 6.9 mmol/L)	4.0	4.3	2.6		5.2			2.9
Sodium (136 – 146 mmol/L)	139	135	139		139			132
Potassium (3.5 – 5.1 mmol/L)	4.2	4.3	4.0		4.1			3.8
Chloride (98 – 107 mmol/L)	105	101	103		104			100
Bicarbonate (19.0 – 29.0 mmol/L)	22.8	23.0	24.4		21.1			20.4
Creatinine (59 – 104 umol/L)	62	90	52		126			50
Liver Function Test								
Bilirubin (7 – 32umol/L)	7		6		7			5
ALT (6 – 66U/L)	316		193		164			193
AST (12 – 42 U/L)	1183		499		479			723
ALP (39 - 99 U/L)	72		61		59			59
GGT (6 – 42 U/L)	28		6		43			21

## Discussion

Exertional rhabdomyolysis, which can be exercise-induced, is a potentially life-threatening medical condition, which has been reported in several medical pieces of literature and has gained increasing public awareness through media discussion. This phenomenon has been observed even in healthy individuals after an intense exercise session [1-5].

Rhabdomyolysis is characterized by the classical triad of myalgia, muscle weakness, and dark tea-colored urine [6]. The myalgia and weakness can be generalized or specific to a muscular group [7]. Clinical presentation can range from asymptomatic elevated creatine kinase to life-threatening presentation of electrolyte imbalances, acute kidney injury, compartment syndrome, and disseminated intravascular coagulation [8].

Delayed-onset muscle soreness (DOMS) is a mild form of rhabdomyolysis, whereby myalgia and muscle weakness are experienced a couple of days after strenuous exercise. This signifies muscle damage but is mild and self-resolving.

Rhabdomyolysis is caused by skeletal muscle damage leading to a breakdown of myocytes, which releases an excessive amount of intracellular protein of creatine kinase (CK), lactate dehydrogenase (LDH), and myoglobin into the systemic circulation [1,9]. These proteins, except myoglobin, are broken down by the reticuloendothelial system. Myoglobin is renally filtered, and it is detectable in the urine when the plasma concentration is above 1.5 mg/dl [10-11]. When the urine concentration of myoglobin is above 100 mg/dl, this pigments the urine to a dark red-brown color [8]. Aciduria and hypovolaemia are two environments that increase susceptibility in developing myoglobinuric acute kidney injury. This causes reduced renal circulation leading to renal vasoconstriction; obstruction via cast formation in the distal convoluted tubules; and direct heme protein nephrotoxicity in the proximal convoluted tubules [12]. Acute kidney failure develops 12-72 hours after the initial insult [8]. The incidence of acute kidney injury is estimated to be between 10% and 30% [8,13].

CK is a sensitive laboratory finding to indicate rhabdomyolysis [14]. This enzyme level rises within the first 12 hours of muscle injury, reaching its peak in one to three days, and then steadily declines over three to five days from the cessation of the muscle injury. The higher the value of peaked CK level is proportional to the predictive risk of developing renal failure [15]. CK levels above 500U/l or more than five times above the limits of normal indicate severe muscle injury and pose an increased risk of renal failure [8,16]. Urine dipstick would test positive for blood in the absence of red blood cells noted on microscopy [17]. In keeping with rhabdomyolysis, the AST and ALT are often elevated (with the rise in AST being more pronounced than ALT), as both of these enzymes can be found in the skeletal muscles.

Electrolyte disturbances are often observed. Calcium and phosphate can form complexes that lead to hypocalcemia. Potassium and phosphate may be elevated from intracellular release and kidney injury. While the conservative approach can be taken with respect to mild phosphate and calcium derangements (especially if the patient is asymptomatic), severe hyperkalemia can lead to cardiac dysrhythmias and subsequently myocardial infarction [8-9]. Intravenous volume resuscitation is the first line of management to enhance renal perfusion and hence minimizing kidney injury. Up to 25% of patients with rhabdomyolysis have hepatic dysfunction secondary to proteases that are released from the myolysis [8].

Exercise rhabdomyolysis is commonly observed in patients after the participation of an unaccustomed exercise regime. This could be individuals who exercise regularly but increased their workout intensity, changed their type of training regime, or trained an undertrained muscle group. In individuals who are less climatized to routine exercise, the acute increase in workout intensity and exerting oneself at the start of a training program put them at risk of developing exercise rhabdomyolysis. When guided by a personal trainer or coach, the trainee is at higher risk of overexertion [18]. Exercises focusing on slow repetitive eccentric contractions cause the contraction of a lengthening muscle, results in an increased strain of the muscle leading to muscle damage and therefore rhabdomyolysis [1].

When comparing resistance versus endurance exercise in leading to rhabdomyolysis, aerobic exercises usually have a lower risk. However, several pieces of literature have observed this phenomenon in marathon runners and increasingly in indoor cycling [19-21]. 

The use of a percussion massage gun post-workout has also been linked to rhabdomyolysis in a reported case report [22]. The repetitive percussion could cause microtear and damage to the muscle leading to rhabdomyolysis.

Indoor cycling, as known as Spinning®, has gained increased popularity in the fitness industry over the last decade. Participants ride on modified stationary bikes to the beat of a specially choreographed music playlist. In some spin studios, this loud music is accompanied by the dimming of the main studio lights and adding of light-emitting diode (LED) light effects. The ambiance pairing of music and dim lights enhances pleasure and reduces the perceived tiredness and hence enables participants to persevere through the workout [23].

Studio instructors tailor the intensity of the session based on the resistance of the bikes, cadence, coordinated hand positions and movements, and change body position (ie, sitting or standing). Spinning is considered a high-intensity cardiovascular workout, with high energy expenditure. This level of intensity should be undertaken with caution in those with a sedentary lifestyle, elderly, or with underlying cardiac health issues [24].

In such strenuous exercise, participants are at risk of dehydration, especially in a hot humid environment like Singapore where our case series are observed. Under-hydration reduces the renal ability to excrete the myoglobin and nephrotoxic byproduct of myocyte breakdown, with subsequent cast formation leading to obstruction. Reduced renal perfusion causes vasoconstriction leading to ischemia-reperfusion, which consequently contributions to acute renal failure [1,12]. Hot and humid conditions also cause the diversion of blood flow from vital organs to the skin and skeletal muscles. Diverted blood flow from the kidney leads to little to no urine due to reduced glomerular filtration rate, which further encourages the development of acute renal failure [25]. Despite being a common precipitating factor for rhabdomyolysis, however, it is not a prerequisite [26]. On the contrary, overhydration can also lead to rhabdomyolysis due to hyponatremia, however, the mechanism is unknown [1].

Other precipitating factors of rhabdomyolysis include exercising with a concomitant viral illness, certain medications or drugs (for example, statin, anticholinergics, anabolic steroids, amphetamines), consumption of dietary supplements (for example, creatine, caffeine), and alcohol intake [1,27]. Statins are an effective medication used in hyperlipidemia that antagonizes 3-hydroxy-3 methylglutaryl co-enzyme A (HMG-CoA) reductase. It is a generally safe medication but one of the known side effects is myalgia, which can be observed in 5% of users. Approximately 0.1% develops rhabdomyolysis. When coupled with exercise, raised CK levels are recorded even in athletes [28].

Some genetic predisposing risks include sickle cell anemia and inborn errors of metabolism causing metabolic myopathies [1]. In patients with inborn errors of metabolism, there is impaired oxidation of fat, amino acid, mitochondrial, or carbohydrate. This predisposes this cohort of patients to develop rhabdomyolysis when performing strenuous activities [29].

Intravenous volume resuscitation is the first line of management to enhance renal perfusion and hence minimizing kidney injury. While the optimal fluid and rate of repletion remain unclear, we can aim resuscitation at the rate of 200-300ml/hour, with careful titration to avoid overhydration and monitoring of acid-base electrolyte balance [6]. The decision on the rate of resuscitation has to be balanced with any other concomitant medical conditions that the patient may have, for example, cardiac or renal disease. Based on the renal function test and electrolytes values, an alternating regime of 0.45% normal saline and Ringer acetate is recommended to avoid hyperchloremic metabolic acidosis and hypochloremic metabolic alkalosis, respectively [9]. Strict fluid balance monitoring are recommended, with the aim of urine output of 200-300 ml/hr [30]. Renal function tests and electrolytes should be monitored regularly and actively corrected if the presence of derangement. Hyperkalaemia, hyperphosphatemia, and hypocalcemia are often observed and should be reversed. Renal dialysis is only resorted to if there is recalcitrant hyperkalemia and acidosis despite active fluid resuscitation.

Acute compartment syndrome is a late complication that can be limb-threatening [16]. Classical symptoms of disproportionate pain, pain on a passive stretch of muscle, paraesthesia, and diminishing pulse, coupled with raised compartmental pressure above 30 mmHg would be present. This is a medical emergency requiring an urgent fasciotomy.

Monitoring of CK levels and symptoms provides an indication of the patient’s recovery trajectory. There is no agreed cut-off value for CK levels when deciding if a patient is fit for discharge. The main indications would be symptoms improvement, no development of complications, and a clear down-trending CK value [9]. Based on the physician's and patient’s comfort levels, patients can be discharged with follow-up in the clinic to continue ongoing monitoring of blood till the levels are back to within normal range of CK values. Patients are recommended to continue oral hydration at home and have complete rest until CK values are back to the normal range. Return of activities should be in phases, initially starting with light activities and no strenuous exercise, and progressing to regular sporting activities and training if the patient tolerates at least one week of light activities without myalgia, weakness, or swelling [17].

From our experience, patients can be safely discharged when the CK values are clearly downward trending and the patient’s symptoms have resolved. As observed in our cohort of patients, all the patients also have raised transaminases. These subsequently resolved once the patient recovers from the rhabdomyolysis. We would advocate for a follow-up clinic review to repeat the CK levels and liver function test to look for resolution. The patient can be safely discharged from medical follow-up once the CK level falls below 1000 prior to discharge.

## Conclusions

Exercise rhabdomyolysis is a potentially life-threatening medical condition, which can easily be prevented with the right education and awareness. Our article highlights the relation between rhabdomyolysis in post-spin patients presenting with the triad of myalgia, muscle weakness, and dark tea-colored urine. Early diagnosis with the measure of creatinine kinase can enable swift management with aggressive intravenous hydration to provide a good prognosis.

It is important to highlight to the public the observed association of spin-induced rhabdomyolysis and spin-naïve participants. We have compiled some pointers for the public to consider to prevent spin-related rhabdomyolysis: Good fluid and electrolyte replacement before, during, and post-workout; Intermittent water breaks during the workout; Good warm-up and cool-own during the spin session; Self-awareness of level of exertion especially when new to spin; Encouraging new participants to inform the instructor that they are new to the class; Avoiding going at low intensities at high cadence; Slowing down the speed when required; Considering the temperature of the spin studio especially in humid countries; Avoiding alcohol peri-spin class; Avoiding creatine and anabolic steroids; avoiding the use of nonsteroidal anti-inflammatory drugs (NSAIDs); Avoiding sauna post-workout; Being aware of the risk in patients on statins and drugs (statin, cholinergic); Avoiding the use of a percussion massage gun; Having adequate sleep and rest post-workout to recover; and Having adequate intervals of a few days between intense workout sessions to allow your body to fully recover.
